# Mechanisms Involved in the Binding of Thymocytes to Rat Thymic Dendritic Cells

**DOI:** 10.1155/1996/18312

**Published:** 1996

**Authors:** Miodrag Čolić, Vesna Ilić, Miloš D. Pavlović, Takuya Tamatani, Masayuki Miyasaka

**Affiliations:** 1 Institute of Medical Research Military Medical Academy Crnotravska 17 Belgrade Serbia and Montenegro; 2 Department of Immunology Tokyo Metropolitan Institute of Medical Science 3-18-22, Hon-Komagome Bun Kyo Tokyo 113 Japan

**Keywords:** Thymic dendritic cells, thymocytes, adhesion molecules, rosettes, signaling

## Abstract

The effects of monoclonal antibodies (mAbs) to cell-surface molecules, divalent cations, and various cell-signaling and metabolic inhibitors on the binding of thymocytes to rat thymic dendritic cells (TDC) were studied using a rosette assay. It was found that TDC/thymocyte adhesion was stronger and faster at 37°C than at 4°C. Flow cytometric analysis demonstrated that bound thymocytes were predominantly CD4^+^CD8^+^ and CD4^+^CD8^-^, but in comparison to the phenotype of whole thymocytes, they were enriched in the mature TCRαβ^hi^ subset. The binding of thymocytes to TDC at 37°C was almost completely dependent on Ca^2+^ and Mg^2+^ and partly on an intact cytoskeleton and calmodulin-dependent protein kinase. The adhesion was independent of new protein synthesis and the activities of protein kinases A and C, tyrosine kinases, as well as phosphotyrosine protein phosphatases. The TDC/thymocyte adhesion at 37°C was partly blocked by anti-LFA-1 (WT.1), anti-CD18 (WT.3), and anti-ICAM-1 (1A29) mAb. MAbs to class II MHC (OX-3 and OX-6), CD4 (W3/25), CD8 (OX-8), and αβTCR (R73) stimulated the adhesion via an LFA-1-dependent pathway, whereas an anti-CD45 mAb (G3C5) stimulated the rosette formation independently of LFA-1. MAbs to CD2 (OX-34), CD11b (ED7), CD11b/c (OX-42), and class I MHC (OX-18) were without significant effects on the adhesion process.


*Developmental Immunology*, 1996, Vol 5, pp. 37–51
Reprints available directly from the publisher
Photocopying permitted by license only

© 1996 OPA (Overseas Publishers Association)
Amsterdam B.V. Published in The Netherlands
by Harwood Academic Publishers GmbH
Printed in Singapore

### Mechanisms Involved in the Binding of Thymocytes to Rat Thymic Dendritic Cells

MIODRAG ČOLIĆ,*[†] VESNA ILIĆ,[†] MILOŠ D. PAVLOVIĆ,[†] TAKUYA TAMATANI,[†] and MASAYUKI MIYASAKA[‡]

[†]*Institute of Medical Research, Military Medical Academy, Crnotravska 17, Belgrade, Yugoslavia*
[‡]*Department of Immunology, Tokyo Metropolitan Institute of Medical Science, 3-18-22, Hon-Komagome, Bun Kyo, Tokyo 113, Japan*

The effects of monoclonal antibodies (mAbs) to cell-surface molecules, divalent cations, and various cell-signaling and metabolic inhibitors on the binding of thymocytes to rat thymic dendritic cells (TDC) were studied using a rosette assay. It was found that TDC/thymocyte adhesion was stronger and faster at 37°C than at 4°C. Flow cytometric analysis demonstrated that bound thymocytes were predominantly $CD4^+CD8^+$ and $CD4^+CD8^-$, but in comparison to the phenotype of whole thymocytes, they were enriched in the mature $TCR\alpha\beta^{hi}$ subset. The binding of thymocytes to TDC at 37°C was almost completely dependent on $Ca^{2+}$ and $Mg^{2+}$ and partly on an intact cytoskeleton and calmodulin-dependent protein kinase. The adhesion was independent of new protein synthesis and the activities of protein kinases A and C, tyrosine kinases, as well as phosphotyrosine protein phosphatases. The TDC/thymocyte adhesion at 37°C was partly blocked by anti-LFA-1 (WT.1), anti-CD18 (WT.3), and anti-ICAM-1 (1A29) mAb. MAbs to class II MHC (OX-3 and OX-6), CD4 (W3/25), CD8 (OX-8), and αβTCR (R73) stimulated the adhesion via an LFA-1-dependent pathway, whereas an anti-CD45 mAb (G3C5) stimulated the rosette formation independently of LFA-1. MAbs to CD2 (OX-34), CD11b (ED7), CD11b/c (OX-42), and class I MHC (OX-18) were without significant effects on the adhesion process.

KEYWORDS: Thymic dendritic cells, thymocytes, adhesion molecules, rosettes, signaling.

### INTRODUCTION

Thymic dendritic cells (TDC) represent minor, but functionally very significant component of the thymic microenvironment (Hamblin and Edgeworth, 1988). They are derived from the bone marrow and selectively colonize cortico-medullary region and medulla (Barclay and Mayrhofer, 1981; Duijvestijn et al., 1984; Kampinga and Aspinal 1990). TDC are believed to participate in the maturational process of thymocyte development (Kyewski, 1988), intrathymic presentation of non-major histocompatibility complex (non-MHC) antigens (Kyewski et al., 1986), clonal deletion of autoreactive thymocytes (Carlow et al., 1992), or clonal amplification of mature medullary thymocytes (Landry et al., 1990). However, how they influence these intrathymic events is still being debated.

Enzyme digestion of thymic stroma enables isolation of different complexes composed of non-lymphoid cells and thymocytes (Kyewski et al., 1982). TDC are also rosette-forming cells (Kyewski et al., 1982; Landry et al., 1990; Shortman and Vremec, 1991). It is postulated that these close cell–cell contacts are responsible for most of TDC functions (Adkins et al., 1986; Kyewski, 1988). Although interactions between peripheral dendritic cells (DC) and T lymphocytes have been extensively explored, little is known about the mechanisms involved in TDC/thymocyte binding. This question is addressed in the present work using an original system for isolation of rat TDC.

### RESULTS

### Dynamics of Thymocyte Adhesion to Rat TDC

To study adhesion characteristics of rat TDC, we first used an alternative method for their isolation and

*Corresponding author.

purification (Ilić et al., in press). This included isolation of low-density cells from thymocyte suspension over a Nycodenz gradient (density 1.078 g/cm$^3$; osmolarity 390 mOsm) and their subsequent cultivation with 20% TE-R 2.5 + HT supernatant obtained by cocultivation of a medullary epithelial cell line (TE-R 2.5) (Čolić et al., 1992) and hydrocortisone-resistant thymocytes. This procedure allowed the recovery of TDC of relative high purity (more than 80%), their differentiation and expression of most markers characteristic for rat TDC *in situ*, such as MHC class I and class II molecules, CD45, CD25, LFA-1 (CD11a/CD18), ICAM-1 (CD54), and OX-44 (CD53). Certain TDC subsets expressed CD11b and thymocyte markers Thy1, CD2, CD4, and CD8. These cells were functionally very active in inducing strong proliferation of autologous thymocytes depleted of endogenous accessory cells even without any additional stimuli (Ilić et al., in press).

Such prepared TDC were incubated with syn-

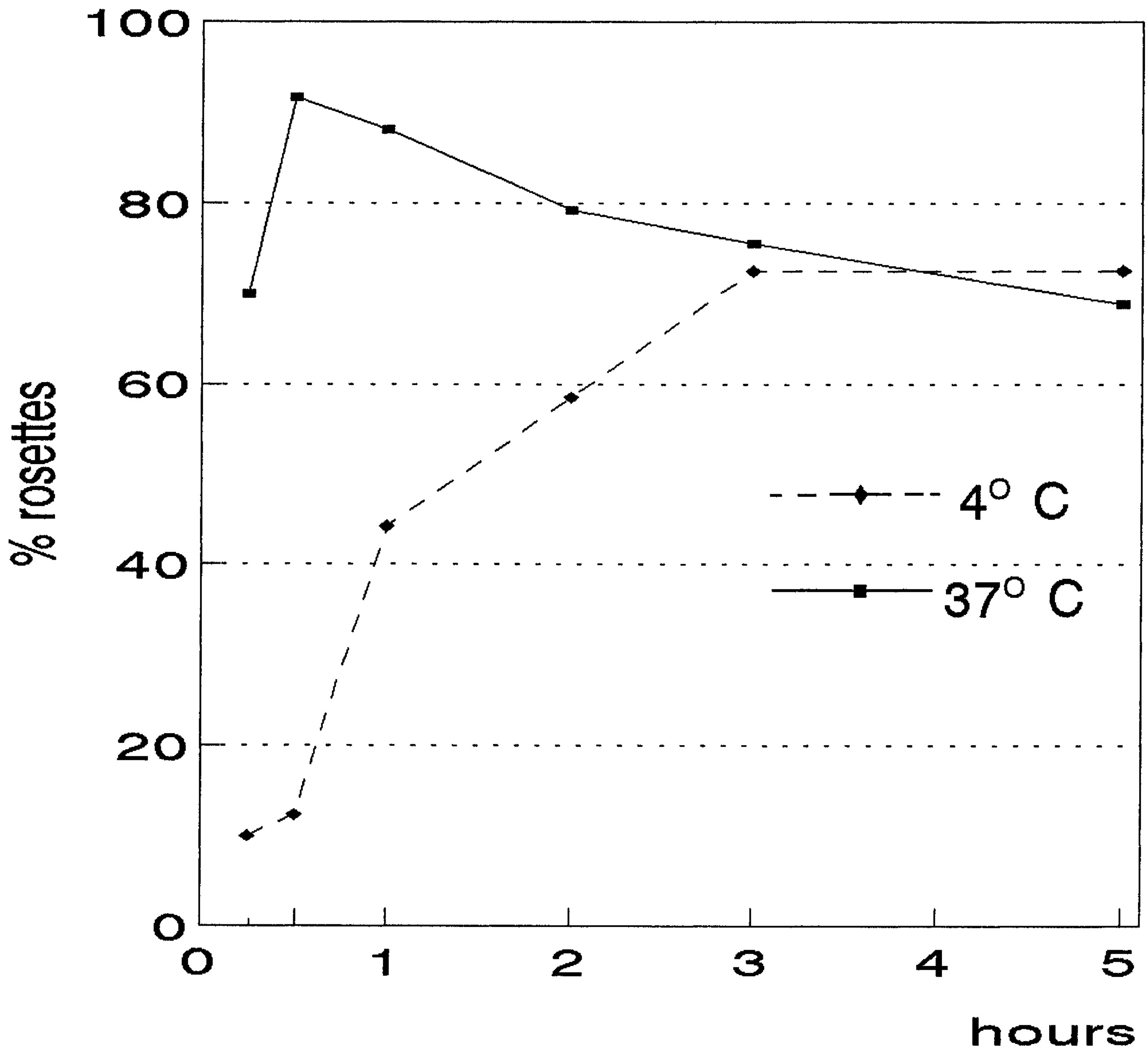

FIGURE 1. Dynamics of rosette formation between TDC and thymocytes. TDC were mixed with thymocytes (ratio 1:20) and incubated in Terasaki plates (hanging drop assay) for different periods of time both at 37 or 4°C. The percentages of TDC-forming rosettes from one of three similar experiments are given.

geneic AO rat thymocytes (ratio 1:20) both at 37 and 4°C using a hanging-drop assay in Terasaki plates. After different periods of time, the percentage of TDC-forming rosettes were calculated. The results presented in Fig. 1 show that most TDC-formed rosettes with thymocytes at 37°C. The binding was very strong because mostly large cell clusters were observed (Fig. 2). The adhesion process was also very fast reaching the maximum as early as after 30 min of cell incubation and slightly decreased thereafter (up to 5 hr). In contrast, the initial binding (30 min) at 4°C was very low. After that, it progressively increased (up to 3 hr) reaching almost the same values as those observed at 37°C (Fig. 1).

### Phenotypic Characteristics of Thymocytes Bound to TDC

We next tested the phenotype of thymocytes bound to TDC by flow cytometry. Purification of rosettes, detachment of thymocytes from TDC, and staining procedure are described in Materials and Methods.

Thymocytes were easily identified by appropriate gating. The results presented in Fig. 3 and Table 1 show that TDC predominantly clustered with $CD4^+CD8^+$ and $CD4^+CD8^-$ thymocytes. However, the percentage of $CD4^+CD8^+$ cells was lower, whereas

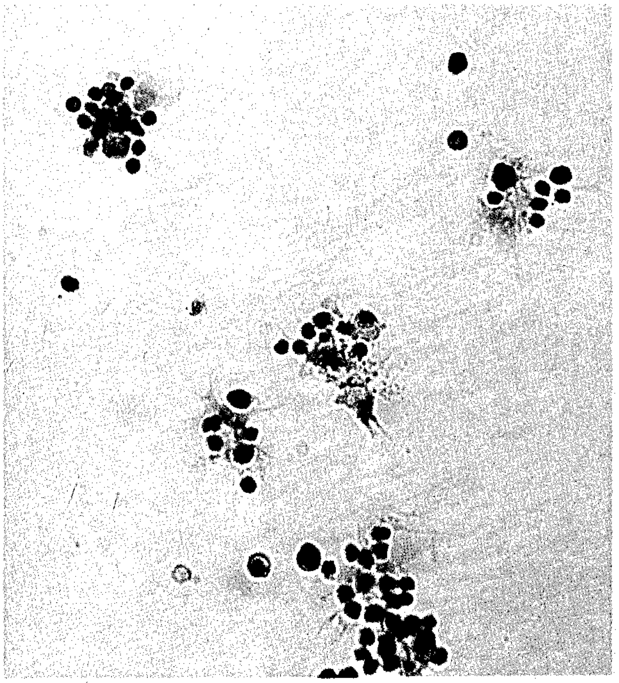

FIGURE 2. TDC rosettes stained with hematoxylin-eosin. TDC rosettes were formed by incubating TDC and thymocytes (ratio 1:20) for 30 min at 37°C as described in Materials and Methods. Before staining rosettes were purified by brief centrifugation (50 *g* for 1 min at 4°C) over FCS and incubated for 20 min on PLL-coated glass slides to allow their better attachment and spreading, followed by 0.1% glutar-aldehide fixation. Magnification ×320.

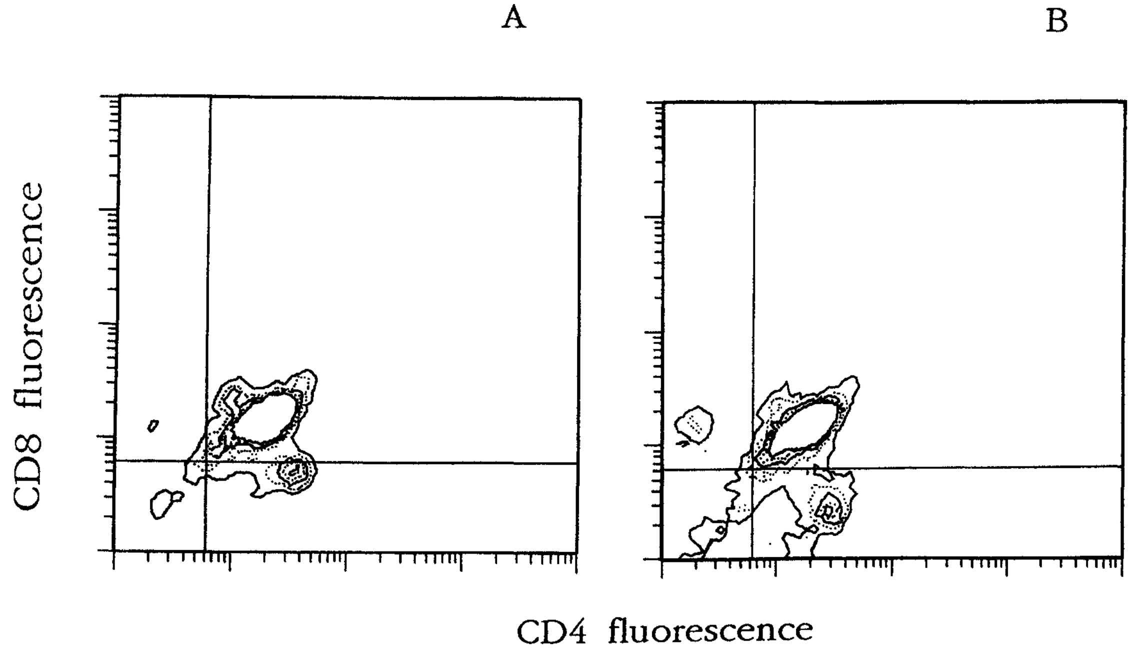

FIGURE 3. Flow cytometric analysis of thymocyte subsets defined by the expression of CD4 and CD8 among total thymocytes (A) and thymocytes bound to TDC *in vitro* (B). The formation of rosettes, detachment of thymocytes from TDC, and staining of thymocytes with mAbs were as described in Materials and Methods. Thymocytes were analyzed on a FACScan flow cytometry by appropriate gating. Representative profiles of CD4 and CD8 expression are given.

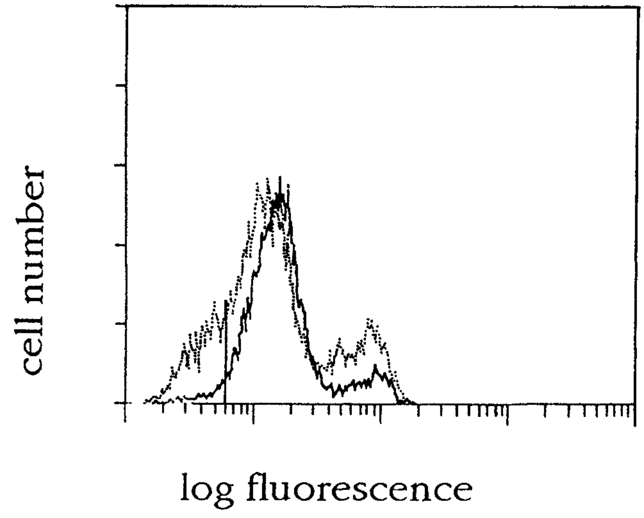

FIGURE 4. Expression of αβTCR on total thymocytes (solid line) and thymocytes bound to TDC *in vitro* (dotted line). Thymocytes were prepared and stained by R 73 mAb as described. The histograms are representative of three experiments with similar results. The vertical bar represents the level of nonspecific staining using PBS and secondary antibody as control.

the percentage of $CD4^+CD8^-$ cells was higher than the values of these cells subsets in the whole thymocyte population. The conclusion that thymocytes bound to TDC were enriched in more mature thymocytes was drawn by analyzing the fluorescence profile of thymocytes stained by an anti-αβTCR antibody (R73). Figure 4 and Table 1 show that among adherent thymocytes (compared to whole thymocytes), a higher percentage of the $\alpha\beta TCR^{hi}$ subset was observed.

### Effect of Bivalent Cations on Rosette Formation between TDC and Thymocytes

The next experiments were designed to study the mechanisms involved in TDC/thymocyte adhesion. We first studied the effect of bivalent cations ($Ca^{2+}$ and $Mg^{2+}$) in the rosette formation after 30 min of cell incubation at 37°C. Figure 5 shows that $Ca^{2+}$- and $Mg^{2+}$-free HBSS medium only partly suppressed thymocyte binding to TDC. The separate addition of $Ca^{2+}$ and $Mg^{2+}$ ions to HBSS medium partly restored thymocyte binding to TDC. If these cations were added simultaneously, the percentage of rosettes did not significantly differ from the values obtained using the classical medium (RPMI+10% FCS).

The effect of $Ca^{2+}$ and $Mg^{2+}$ chelators (EDTA and EGTA) was also tested both at 37 and 4°C. Figure 6 shows that these agents in a dose-dependent manner (except at 4°C, after 3 hr) suppressed rosette formation. The percentage of inhibition was almost equal independently of incubation temperature. However, the inhibition decreased with prolonged incubation (3 hr).

### Signaling Pathways Involved in TDC/Thymocyte Adhesion

Signaling pathways involved in the rosette formation between TDC and thymocytes were studied using various types of metabolic inhibitors. Figure 7 shows that cytochalasin B (an inhibitor of microfilament formation) partially decreased the percentage of rosettes at 37°C (30 min). Similar results were obtained after 3 hr of cell incubation (not shown). As expected, cycloheximide (an inhibitor of protein synthesis) did not influence the TDC/thymocyte binding. We also treated TDC and thymocytes with various protein kinases inhibitors at 37°C. H7 (a PKA and PKC inhibitor) and genistein (a tyrosine kinase inhibitor) did not influence the adhesion process. In contrast, W7 (specific inhibitor of calmodulin-dependent protein kinase) was partly inhibitory. Similar inhibition was seen after 3 hr (not shown). The adhesion was not modified by using

TABLE 1
Phenotype of Rat Thymocytes Bound to TDC[a]

| Phenotype | Total thymocytes (%) | Bound thymocytes (%) |
|---|---|---|
| $CD4^-CD8^-$ | 2.1±0.5 | 2.1±1.2 |
| $CD4^+CD8^+$ | 83.8±3.6 | 75.7±5.6 |
| $CD4^+CD8^-$ | 9.2±0.6 | 17.5±2.6[b] |
| $CD4^-CD8^+$ | 4.7±2.8 | 4.7±2.5 |
| α/βTCR low/interm. | 52.2±6.1 | 48.3±4.1 |
| high | 12.0±3.2 | 23.5±3.6[b] |

[a]The formation of rosettes, detachment of thymocytes from TDC, and staining of thymocytes with mAbs were as described in Materials and Methods. Thymocytes were analyzed on a FACScan flow cytometer by appropriate gating. Values (mean ± SD from 3 different experiments) are given as percentages of a particular thymocyte subset.
[b]$p < 0.001$ compared to corresponding values of total thymocytes.

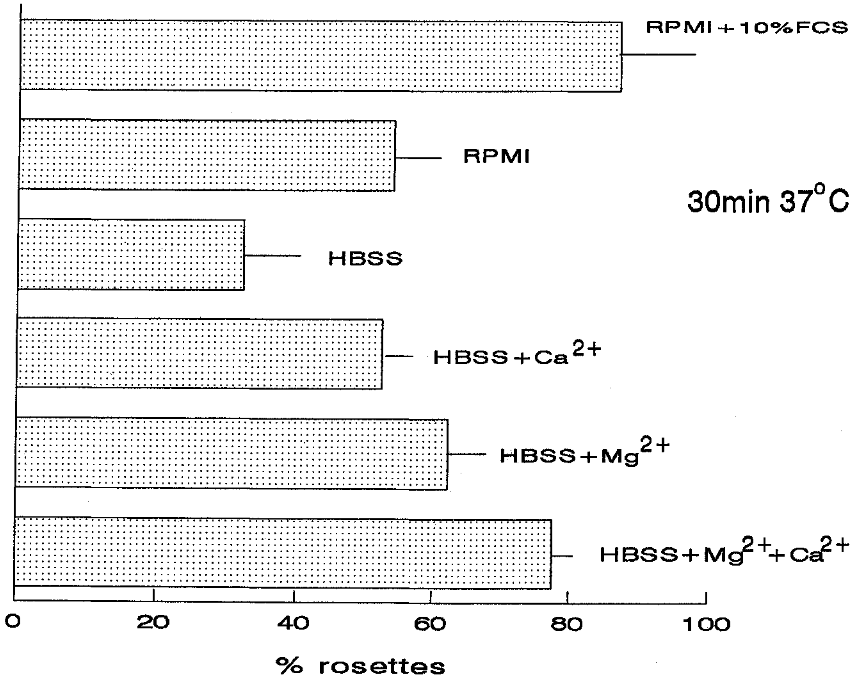

FIGURE 5. Effect of bivalent cations on thymocyte binding to TDC. Thymocytes were mixed with TDC in different media. $Ca^{2+}$ and $Mg^{2+}$ were used at concentration of 5 mM. Cells were incubated for 30 min at 37°C. The results are presented as the mean percentages of rosettes ± SD from 3 different experiments.

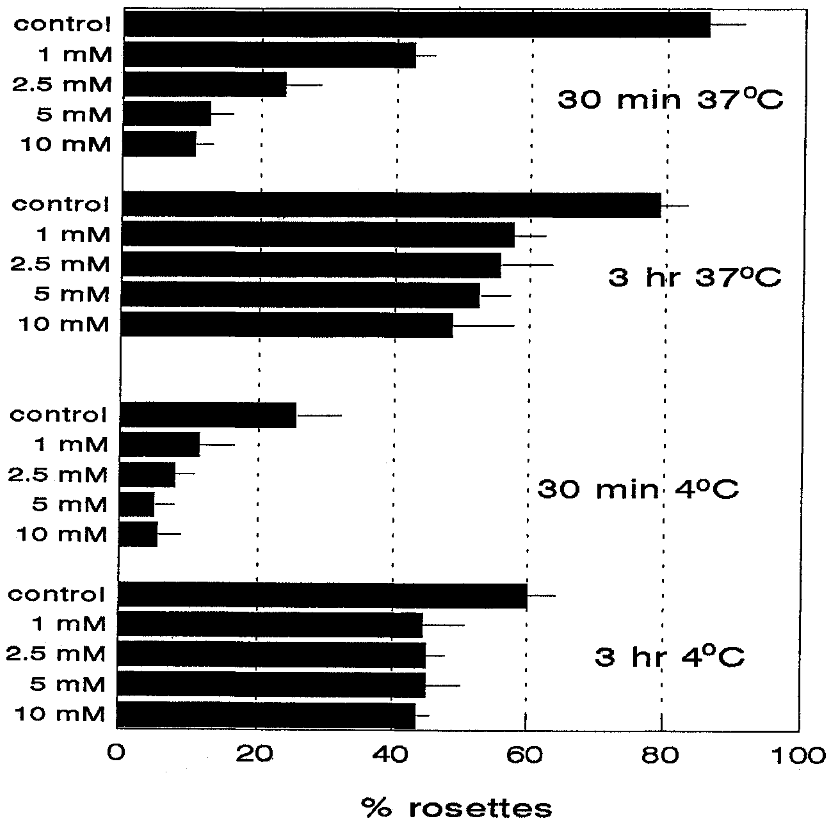

FIGURE 6. Effect of bivalent cation chelators (EGTA + EDTA) on thymocyte binding to TDC. Thymocytes and TDC were incubated in RPMI/10% FCS at 4 or 37°C for 30 min or 3 hr. EDTA and EGTA were added at concentrations of 1–10 mM. The results are presented as mean of rosette percentages ± SD from 3 different experiments.

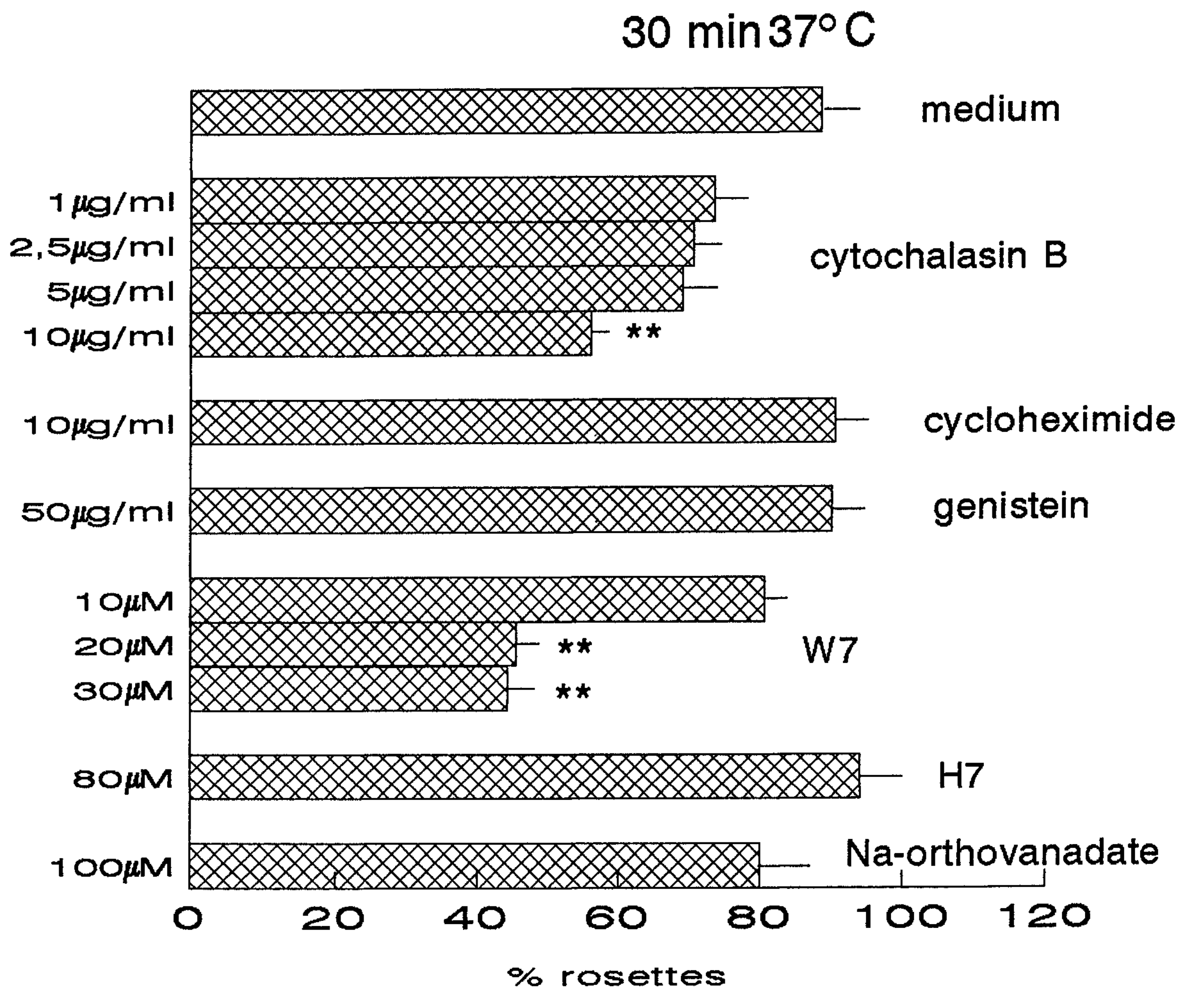

FIGURE 7. Effect of various metabolic and cell-signaling inhibitors on thymocyte binding to TDC. TDC and thymocytes were preincubated with different inhibitors for 30 min at 37°C before mixing as described. Cells were then incubated with inhibitors for additional 30 min at 37°C. The results are presented as the mean percentages of rosettes ± SD from 3 different experiments. ** = $p < 0.001$ compared to control (RPMI/10% FCS medium).

sodium orthovanadate (an inhibitor of protein phosphotyrosine phosphatases).

### Surface Cell Molecules Involved in TDC/ Thymocyte Binding

The final aim of this work was to examine the role of cell-surface molecules in TDC/thymocytes adhesion by using mAbs directed to several rat adhesion molecules. Figure 8 shows the effect of mAbs to β2 integrins, ICAM-1, and CD2 on TDC/thymocyte binding. The results demonstrated that of the mAbs, WT.1 (anti-LFA-1), WT.3 (anti-CD18), and 1A29 (anti-ICAM-1) mAbs were partly inhibitory after 30 min. WT-3 had the strongest effect (approx. 55% inhibition) that was not further potentiated using combination of WT.1 and WT.3. Figure 8 also shows that inhibitory effects of WT.1 and WT.3 were lesser after 3 hr of cell incubation. At that time, point 1A29 was not inhibitory. The mAbs to CD11b (ED7) or CD11b/c (OX-42) as well as CD2 (OX-34) did not significantly modify the adhesion. The inhibitory effect of OX-34 mAb on TDC/thymocyte adhesion was also not seen under condition when the main pathway (LFA-1/CD18) was blocked using a combination of WT.1, WT.3, and OX-34 mAbs.

A lot of mAbs stimulated TDC/thymocyte binding. They were OX-3 and OX-6 (both directed to class II MHC molecules), R73 (anti-αβTCR), W3/25 (anti-CD4), OX-8 (anti-CD8), and G3C5 (anti-CD45 framework) antibody (Fig. 9). The stimulated adhesion was not only manifested by an increase in the percentage of rosettes, but also by a significant enlargement of their sizes.

The stimulatory effect of anti-αβTCR, anti-CD4, and anti-CD8 was visible during the first adhesion phase (30 min), whereas the effect of OX-3 and OX-6 mAbs was observed after 3 hr of cell incubation. G3C5 mAb stimulated TDC/thymocyte binding in both adhesion phases. OX-18 mAb, directed to class I MHC, was without significant effect in this process. We further tested whether stimulatory effects of these mAbs were mediated by LFA-1. The results presented in Fig. 9 show that, except for G3C5, preincubation of thymocytes with WT. 1 abrogated the stimulated adhesion of all these mAbs.

### DISCUSSION

This work was designed to study the mechanisms involved in the adhesion between TDC and thymocytes. We started the experiments using a modification of the procedure for isolation of rat TDC. It involved selective centrifugation of whole thymocyte suspension over a Nycodenz gradient (14.5%, osmolarity 390 mOsm) and further cultivation of low-density cells (TDC purity about 30%) for 3 days in medium supplemented with the TE-R 2.5 + HT supernatant. This supernatant was prepared by cocultivation of a rat medullary thymic epithelial cell line (TE-R 2.5) (Čolić et al., 1992) with hydrocortisone-resistant thymocytes. Cytokines and other soluble factors derived from these cells promoted significant survival of TDC in culture and their morphological and phenotypical differentiation to the cells expressing most characteristics of TDC *in situ*. At the same time, TDC purity significantly increased (more than 80%) due to apoptosis of contaminating thymocytes and selective attachment of thymic MΦ and monocytelike cells to plastic. All these properties of TDC were presented in our recent work (Ilić et al., in press).

Using such prepared cells, we found that rat thymocytes strongly bound to TDC and formed large clusters (rosettes). Attached thymocytes were enriched in the $\alpha\beta TCR^{hi}$ subset (predominantly $CD4^+CD8^-$), but a large proportion of thymocytes were phenotypically immature, $CD4^+CD8^+$ cells. Such an *in vitro* analysis of thymocyte phenotype has not been performed. However, the results are similar to those of Shortman and Vremec (1991), who analyzed by flow cytometry the phenotype of thymocytes bound to mouse TDC, immediately after *in vivo* isolation of the rosettes. They found that the TDC rosettes were enriched in thymocytes expressing high levels of surface TCR and CD3, and these included both $CD4^+CD8^-CD3^{++}$ and $CD4^-CD8^+CD3^{++}$ mature thymocytes. Both these and our results are in agreement with the topographical localization of TDC *in situ* (cortico-medullary zone and medulla) in which final differentiation and maturation processes of thymocytes occur (Barclay and Mayrhofer, 1981; Hamblin and Edgeworth, 1988). The finding that $CD4^+CD8^+\alpha\beta TCR^{low}$ (immature phenotype) thymocytes showed a similar incidence in total thymocyte pool as the population isolated from TDC after binding, suggests that the immature thymocytes in the cortex have already developed the potential to bind TDC that they may encounter later on during their intrathymic sojourn. Physiologically, thymocytes with this phenotype can be localized in the cortico-medullary zone where they may come into close contact with TDC. The

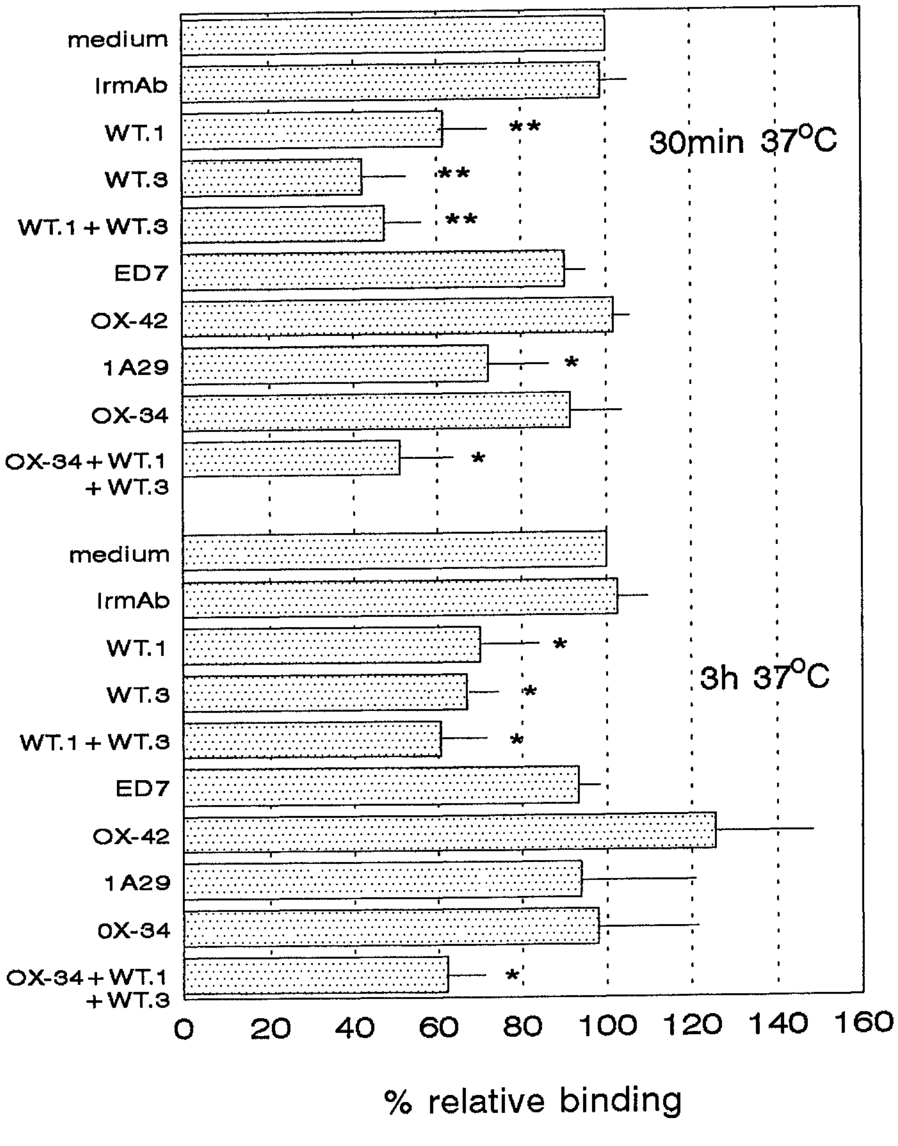

FIGURE 8. Effect of mAbs to rat adhesion molecules on thymocyte binding to TDC. Thymocytes and TDC were prepared as described in Materials and Methods. Before mixing, thymocytes were preincubated for 30 min at 4°C with mAbs WT.1, WT.3, OX-34, combination of these mAbs or BH1 (an irrelevant [Ir] mAb) and TDC were preincubated with 1A29, ED7, OX-42 mAbs or BH1, all at the concentrations of 10 μg/ml. Values (mean ± SD from three to four different experiments) are given as percentage relative binding to control (medium without mAb) (100% relative binding). * = $p < 0.01$; ** = $p < 0.001$ compared to Ir mAb.

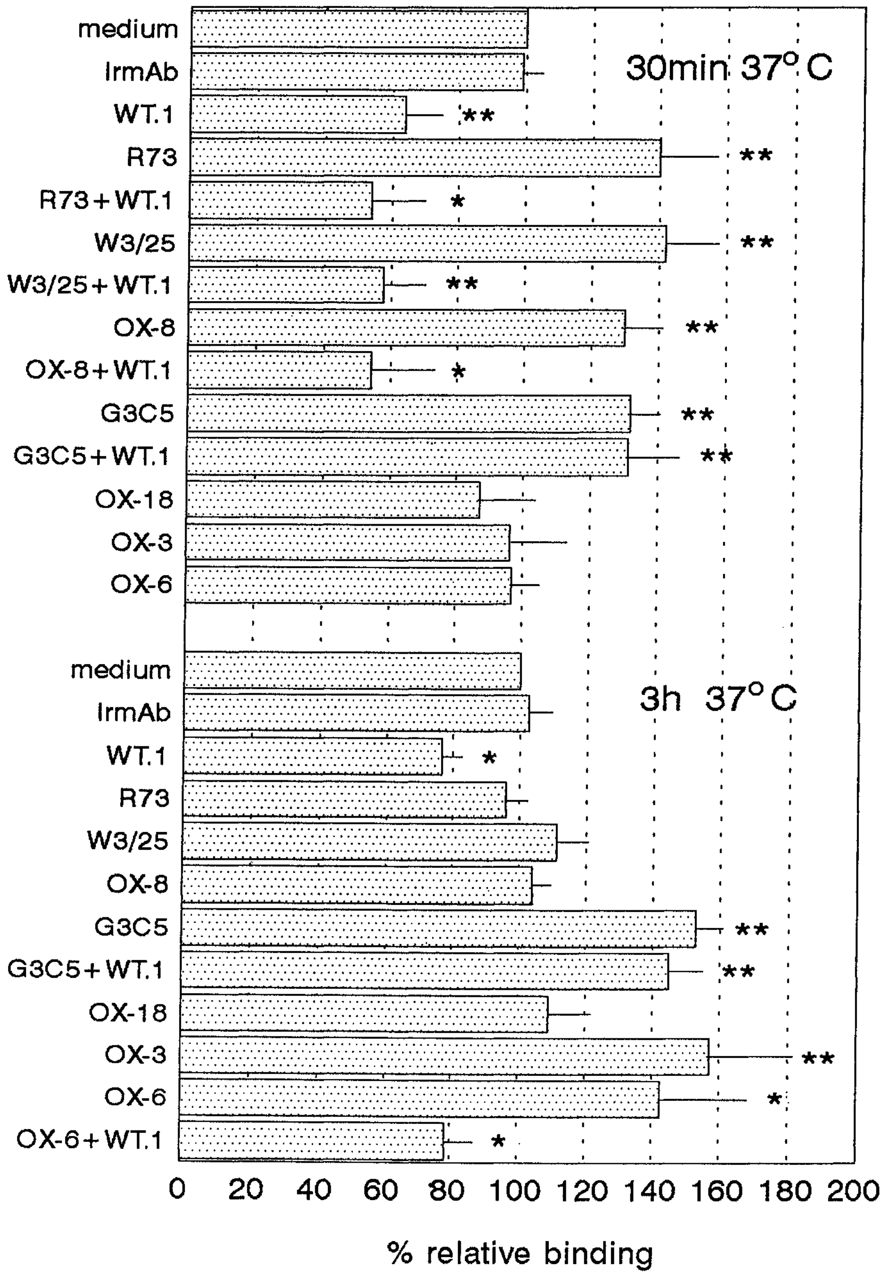

FIGURE 9. Effect of mAbs reactive with rat cell-surface antigens on thymocyte binding to TDC. Thymocytes and TDC were prepared as described in Materials and Methods. Before mixing, thymocytes were preincubated for 30 min at 4°C with mAbs R 73, W3/25, OX-8, G3C5, WT.1, IrmAbs (BH1 or BH2 mAb), or WT.1 in combination with these mAbs, and TDC were preincubated with OX-3, OX-6, OX-18 mAbs, or BH1, all at the concentration of 10 μg/ml. Values (mean ± SD from three to four different experiments) are given as percentage relative binding to control (medium without mAb) (100% relative binding). * = $p < 0.01$; ** = $p < 0.001$ compared to Ir mAb (BH1). Values for BH2 were almost the same as BH1 (data not shown).

contact of TDC with $CD4^+CD8^+$ is also possible in the medulla. Using double immunofluorescence, we found that 15–20% of thymocytes in the medulla of AO rat thymus were $CD4^+CD8^+$ (Čolić, unpublished observation). Some immunohistological investigations also showed preferential contacts of $CD4^+$ T cells with DC *in situ* (Janossy et al., 1980), but more precise dual immunolabeling was not performed to demonstrate whether they are double ($CD4^+CD8^+$) or single ($CD4^+CD8^-$) positive. We think that TDC have different impact on distinct thymocyte subsets (induction of apoptosis of $CD4^+CD8^+$ cells; stimulation of proliferation of $CD4^+CD8^-$ cells). This hypothesis is currently being tested in our laboratory.

The main aspect of this work concerned the mechanisms involved in the binding of thymocytes to TDC. Such studies are scarce, but it is well known that DC isolated from peripheral lymphoid tissues efficiently cluster T cells, a phenomenon that is not dependent on antigen-specific interaction, but rather engagement of various adhesion molecules (Inaba et al., 1989; King and Katz, 1989). Our findings indicate that the LFA-1/ICAM-1 interaction plays a key role in the early adhesion phase (30 min) between thymocytes and TDC, when the adhesion process was maximal. Inhibitory effects of mAbs against these molecules decreased after prolonged incubation (3 hr). This is in accordance with previous studies in other cells systems showing the role of the LFA-1 molecule in the stabilization of the early rapid adhesion process (Dustin and Springer, 1989; Lepesant et al., 1990). Scheeren et al. (1991) demonstrated that clustering of human peripheral blood DC with T lymphocytes was predominantly LFA-1/ICAM-1-dependent. However, in contrast to our results, the inhibitory effect of applied mAbs was seen after 4 hr of cell incubation, at the time point of maximal adhesion. The difference could be explained by different dynamics of cell adhesion in these two systems, differences in T cells used (thymocytes vs. peripheral T cells) and probably different phenotypic and functional status of DC.

In our experiments, this adhesion pathway was probably a consequence of the binding of LFA-1 expressed on thymocytes to ICAM-1 expressed on TDC, but an engagement of LFA-1 on TDC with a subset of thymocytes expressing ICAM-1 could be also considered. Namely, in our previous studies, we found that rat TDC express both LFA-1 and ICAM-1 (Ilić et al., in press), whereas almost all thymocytes and a subset of these cells (20%) are $LFA-1^+$ and $ICAM-1^+$, respectively (Čolić et al., 1994). Our results also demonstrated that anti-LFA-1 and anti-CD18 mAbs had stronger inhibitory effects than anti-ICAM-1 mAb, suggesting that LFA-1/CD18 might use other ligands (ICAM-2 or ICAM-3) (Staunton et al. 1989; Fawcett et al., 1992). However, little is known about their expression on TDC and mAbs to these molecules in rat are not available. The importance of the LFA-1/ICAM-1 pathway in our experiments was also documented by the dependence of cell clustering upon $Ca^{2+}$ and $Mg^{2+}$ ions. It is well known that these cations are necessary for β2 integrin function (Patarroyo et al., 1990; Springer, 1990).

Significance of the LFA-1/ICAM-1 pathway in thymocyte maturation is well documented because mAbs to these molecules block the development of DP thymocytes in fetal thymic organ culture (Fine and Kruisbeek, 1991) and antigen-dependent deletion of DP thymocytes from mice that are transgenic for class I MHC-restricted TCRs (Carlow et al., 1992; Pircher et al., 1993). The latter findings are of special interest because TDC are believed to play a crucial role in negative selection (deletion of autoreactive T cells in the thymus) (Ramsdell and Fowlkes, 1990).

We demonstrated that TDC/thymocyte binding was not completely blocked by anti-LFA-1, anti-CD18, and anti-ICAM-1 mAbs, suggesting that other adhesion molecules are involved.

MAbs against CD11b were not inhibitory, which is in contrast with their effect on clustering between peripheral rat DC and T lymphocytes (Damoiseaux, 1991) or thymocytes and thymic macrophages (MΦ) (Čolić et al., 1994). One possible explanation of the phenomenon is a low expression of this β2 integrin on TDC (Ilić et al., in press). Scheeren et al. (1991) also reported that adhesion between peripheral DC and T cells was partly LFA-1-independent because T cells from leukocyte adhesion deficiency (LAD), which are defective in β2 integrin expression, bound to DC at low rates. We did not identify other adhesion molecules because numerous mAbs against different surface-cell molecules were not inhibitory. It was important to stress that OX-34 (anti-CD2) mAb used in this study as well as other anti-CD2 mAbs (OX-54 and OX-55) (data not shown) did not inhibit rosette formation, suggesting that the CD2/CD48 interaction (Killen et al., 1988) was not probably operative under these experimental conditions. It was demonstrated that the CD2/LFA-3 pathway is not involved in T-cell/blood-DC (Scheeren et al., 1991) binding, which is in contrast to the situation of T-cell adhesion to tonsillar DC, where this pathway has been found to play a role (King and Katz, 1989).

A number of adhesion molecules has been identified on DC such as B7, B7-2, VCAM-1, CD44 (King and Katz, 1989; Scheeren et al., 1991; Caux et al., 1994; Inaba et al., 1994), but their role in binding of thymocytes to TDC has not been studied. We found that mAbs against CD4, CD8, αβTCR, and class II MHC molecules stimulated TDC/thymocyte adhesion. The process was probably not a consequence of a simple crosslinking of mAbs on cell surface because the stimulated adhesion was not seen at 4°C (not shown) and was inhibited by LFA-1 (Fig. 9). In addition, an enhancement of clustering was also observed when both TDC and thymocytes were preincubated with saturated concentrations of the mAbs before cell mixing (data not shown). It could be postulated that under these conditions, both specific receptors and maybe Fc receptors on TDC (if they exist) are occupied precluding the possibility of crosslinking. Many mAbs, with different isotypes (IgG1 or IgM), to rat cell-surface molecules that are abundantly expressed on thymocytes, such as anti-CD2 (Fig. 8) or anti-CD43 and OX-52 (not shown), were not stimulatory. This is another argument favoring the specificity of the observed phenomenon.

The stimulated adhesion could be explained by previous results that showed that signaling through TCR and coreceptor molecule triggered by specific mAbs to these antigens increased affinity of LFA-1 for its ligand (Dustin and Springer, 1989; van Kooyk et al., 1989). The effect was not a consequence of the upregulation of the LFA-1 expression on cell surface, but rather was a result of conformational changes of the LFA-1 molecule. A similar effect of mAbs to CD3, CD4, and CD8 was observed in another system of heterotypic cell adhesion using thymocytes and thymic epithelial cell lines (Lepesant et al., 1990). Certain mAb to class II MHC induced homotypic aggregation of lymphocytes that was also partly LFA-1-dependent (Kansas and Tedder, 1991).

Our preliminary results showed that all these stimulatory antibodies modify intracellular signaling pathways of thymocytes, but their effects on thymocyte proliferation in the presence of TDC are not the same. Namely, R73 stimulated thymocyte proliferation, whereas the others were inhibitory. The results are in agreement with those reported by Xu et al. (1992), who did not show the correlation between the intensity of human blood DC/T lymphocyte clustering and proliferation rates of the T cells.

In contrast to the previous mAbs, an anti-CD45 framework mAb (G3C5) stimulated the adhesion via an LFA-1-independent pathway. This antibody, which has been recently produced in our laboratory (Pavlović et al., manuscript in preparation), induces strong homotyipic aggregation of rat leukocytes. It was also found that certain anti-human CD45 mAbs either increased the size of clusters in culture of DC and human T lymphocytes (Xu et al., 1992) as well as tonsillar T cells and U-937 cells (King et al., 1990) or reduced cluster stability between DC and T lymphocytes (Prickett and Hart, 1990). G3C5 is different from similar proaggregatory anti-CD45 mAbs (Lorenz et al., 1993; Bernard et al., 1994) by its capability to induce the LFA-1-independent adhesion of both resting and activated leukocytes and at the same time to stimulate mitogen-induced T-lymphocyte proliferation and allogeneic MLR. The effect is probably epitop-specific and is not influenced by isotype of the mAb (IgM) (Pavlović et al., manuscript in preparation). The mechanisms involved in the processes are currently investigated Scheeren et al. (1991) also demonstrated that an anti-CD44 mAb enhanced conjugate formation between T cells and blood DC that could not be blocked by anti-LFA-1 mAb.

Our results showed that an intact cytoskeleton and the activity of calmodulin-dependent protein kinase were partly responsible for TDC/thymocyte adhesion because the process was blocked by cytochalasin B and W7, respectively. This is in agreement with the results published for blood DC/T-cell binding (Scheeren et al., 1991). We hypothesize that W7 inhibits cell adhesion by modulating cytoskeleton integrity, because similar observations were published in our recent study dealing with the LFA-1-dependent homotypic aggregation of rat leukocytes (Pavlović et al., 1994).

Blood DC/T-cell adhesion was also partly blocked by H7 (an inhibitor of PKA and PKC) (Scheeren et al., 1991), which was not seen in our experiments. We also found that neither inhibition of protein tyrosine kinases by genistein nor inhibition of protein tyrosine phosphatases by sodium orthovanadate blocked rosette formation. These enzyme inhibitors have not been tested in other similar cell systems.

We also found that the kinetics of rosette formation differ between 4 and 37°C. Both processes were partly dependent on $Ca^{2+}$ and $Mg^{2+}$ ions. At the same time, anti-LFA-1/CD18 mAbs were without significant effect at this temperature (data not shown). At the moment, it is not clear whether this difference is due to difference in binding characteristics and/or dependent on lower-cell motility at 4°C. So this phenomenon needs to be explored in more detail in further experiments.

In conclusion, this work shows that thymocyte adhesion to TDC is a complex and poorly understood process and underlines some differences between our results and those published for T-cell binding to peripheral DC. The differences could be a consequence of specific functions of TDC in thymocyte development, which are attractive for further studies.

### MATERIALS AND METHODS

### Animals

AO rats, both sexes, 8–10 weeks old, bred at the Farm for Experimental Animals (MMA, Belgrade), were used in this study.

### MAbs and reagents

A large panel of mAb was used (Table 2). G3C5 (anti-CD45 framework) (IgM) (Pavlović et al., manuscript in preparation) was produced at the Institute of Medical Research (MMA, Belgrade). Mabs WT.1 (anti-CD11a; IgG2a), WT.3 (anti-CD18; IgG1; Tamatani et al., 1991a) and 1A29 (anti ICAM-1; IgG1; Tamatani and Miyasaka 1990) were produced in Tokyo. All these mAbs recognize "inhibitory" epitopes on the corresponding molecules (Tamatani et al., 1991b). ED7 mAb (IgG1) reactive with rat CD11b (Damoiseaux et al., 1989) was a gift from Dr. C. Dijkstra (Amsterdam) and R73 (anti-αβTCR; IgG1; Hunig et al., 1989) was a gift from Dr. T. Hunig (Wurzburg). OX-18 (anti-class I MHC molecule; IgG1), OX-3 (anti-class II MHC molecule; IgG1), OX-6 (anti-class II MHC molecule; IgG1), OX-42 (anti-CD11b/c; IgG2a), OX-34 (anti-CD2; IgG1), W3/25 (anti-CD4; IgG1), and OX-8 (anti-CD8; IgG1) were obtained from Serotec (UK). For flow cytometric analysis, anti-CD-FITC (Serotec) was used.

The metabolic inhibitors used in this work were Genistein, an inhibitor of tyrosine kinases; H7, an inhibitor of PKA and PKC; W7, an inhibitor of calmodulin-dependent protein kinase; sodium orthovanadate, an inhibitor of phosphotyrosine protein phosphatases; cytochalasin B, an inhibitor of microfilament formation; and cycloheximide, an inhibitor of protein synthesis. All chemicals were obtained from Sigma (USA). Their concentrations used in the experiments were nontoxic for cells, studied by tripan blue dye exclusion.

### Isolation of TDC

Thymic-cell suspension was obtained by teasing thymuses against a steel mesh. Released cells were collected and resuspended in RPMI-1640 medium (Serva, Munich) containing 10% fetal calf serum (FCS) (Flow, Irving, Scotland), 2 mM l-glutamine, and 1% gentamycin with addition of 0.04% EDTA to dissociate thymic clusters. The cell suspension was filtered through a nylon gauze to remove fibrous residue. To enrich this suspension for TDC, the cells resuspended in the same medium ($2 \times 10^8$ cells/3 ml) were layered above 3 ml of Nycodenz gradient (density 1.078 g/cm$^3$ and osmolarity 390 mOsm) and centrifuged at 600 g for 15 min at room temperature. Cells from the interface zone were collected, washed twice, adjusted at $2.5 \times 10^6$ cells/ml and cultivated for 3 days in RPMI/10% FCS medium with addition of 20% TE-R 2.5 + HT supernatant (24-well, flat bottom plates; Flow) in an incubator with 5% $CO_2$. The TE-R 2.5 + HT supernatant was prepared by cocultivating a confluent monolayer of a rat thymic medullary epithelial cell line TE-R 2.5 (Čolić et al., 1992) and hydrocortisone-resistant thymocytes as described (Ilić et al., in press). After this culture period, TDC became nonadherent and formed large clusters. Contaminating MΦ became adherent whereas most thymocytes died. Nonadherent cells

TABLE 2
Characteristics of Monoclonal Antibodies Used in This Study

| mAb | Isotype | Specificity | Source |
|---|---|---|---|
| OX-3 | IgG1 | MHC class II | A |
| OX-6 | IgG1 | MHC class II | A |
| OX-18 | IgG1 | MHC class I | A |
| G3C5 | IgM | CD45 | B |
| 1A29 | IgG1 | CD54 | C[a] |
| WT.1 | IgG2a | CD11a | C[b] |
| WT.3 | IgG1 | CD18 | C[b] |
| ED7 | IgG1 | CD11b | D |
| OX-42 | IgG2a | CD11b/c | A |
| OX-34 | IgG1 | CD2 | A |
| W3/25 | IgG1 | CD4 | A |
| OX-8 | IgG1 | CD8 | A |
| R 73 | IgG1 | αβTCR | E |

A. mAb obtained commercially from Serotec (UK). B. mAb produced at the Institute of Medical Research, MMA (Belgrade) (Pavlovic et al., manuscript in preparation). C. mAb obtained from Dr. M. Miyasaka, Tokyo (Tamatani and Miyasaka, 1990)[a] and (Tamatani et al., 1991a)[b]. D. mAb obtained from Dr. C. Dijkstra, Amsterdam (Damoiseaux et al., 1989). E. mAb obtained from Dr. T. Hunig (Wurzburg) (Hunig et al., 1989).

were collected, resuspended in RPMI/10% FCS medium with 0.04% EDTA, to dissociate clusters, and purified again over a Nycodenz gradient as described before. The purity of such prepared TDC was usually more than 80%.

### Rosette Assay for TDC-Thymocyte Binding

Cultivated TDC ($1 \times 10^4$) were mixed with $2 \times 10^5$ thymocytes (ratio 1:20) in 20 μl of RPMI/10% FCS medium in Terasaki microwell plates and then cultivated in hanging drops at 4 or 37°C for various periods of time. The cells were observed under a light microscope. TDC that bound 4 or more thymocytes were scored as rosettes. For each assay, 200 TDC were counted and each determination was performed in duplicate. The results are given as the mean percentage of rosettes.

For experiments in which we studied signaling pathways involved in rosette formation TDC and thymocytes were preincubated for 30 min at 37°C in RPMI/10% FCS medium with the addition of cytochalasin B (1–10 μg/ml), cycloheximide (10 μg/ml), genistein (50 μg/ml), H7 (80 μM), W7 (10–30 μM), or sodium orthovanadate (100 μM). For statistical analysis (Student *t* test), the percentage of rosettes formed in the presence of a specific inhibitor was compared with that formed in RPMI/10% FCS medium.

For experiments in which we studied effects of bivalent cations on rosette formation TDC and thymocytes were washed in HBSS medium without $Ca^{2+}$ and $Mg^{2+}$ and then incubated in HBSS medium with the addition of 5 mM $CaCl_2$ (Serva or 5 mM $MgCl_2$ (Serva), or with the simultaneous addition of 5 mM $CaCl_2$ and 5 mM $MgCl_2$.

For blocking experiments, TDC were preincubated with OX-3, OX-6, OX-18, 1A29, ED7, or OX-42 mAb, and thymocytes were preincubated with WT.1, WT.3, OX-34, R73, W3/25, OX-8, or G3C5 mAb at 4°C for 30 min. Both TDC and thymocytes were preincubated with irrelevant mAbs BH1 (IgG1) or BH2 (IgM), both against Blastocystis hominis, produced at the Institute of Medical Research (MMA, Belgrade). All antibodies were continuously present during the assay. The results are presented as

$$\% \text{ Relative binding} = \frac{\text{number of rosettes with mAb}}{\text{number of rosettes without mAb}} \times 100$$

For statistical analysis (Student *t* test), the percentage of relative binding in the presence of specific mAb was compared with that using isotype corresponding to irrelevant mAb.

### Flow Cytometry

TDC rosettes formed at 37°C after 45 min were layered over 3 ml of FCS and centrifuged at 50 *g* for 1 min at 4°C. Single cells from the upper layer and the interface zone were discarded. Cell clusters in the sediment were washed and resuspended in PBS with 2% FCS, 0.1% sodium azide, and 0.04% EDTA. This treatment dissociates thymocytes from TDC. Thymocytes were then adjusted at concentration of $2 \times 10^5$ cells/tube and incubated with mAbs diluted in PBS with 2% FCS, 0.1% sodium azide, and 0.04% EDTA for 45 min at 4°C.

Single staining was performed using R73 (anti-αβTCR) mAb followed by a sheep anti-mouse Ig antibody conjugated with FITC (INEP, Zemun, YU). Two-color staining for CD4 and CD8 expression on thymocytes was performed by sequential incubation of the cells with OX-8 mAb, biotin-conjugated mouse anti-rat IgG1 (Serotec), streptavidin-PE (Serotec), and anti-CD4-FITC mAb. All antibodies were diluted in PBS with 2% FCS, 0.1% sodium azide, and 0.04% EDTA.

Stained cells were fixed in 4% formalin and analyzed on a FACScan flow cytometer (Becton Dickinson). TDC were excluded by appropriate gating on the basis of forward scatter vs. side angle scatter profile. Expression of αβTCR is displayed as histograms of green fluorescence (log) vs. number of ($5 \times 10^3$) cells. Results of two-color staining are displayed as histograms of green fluorescence (log) vs. red fluorescence (log).

Background fluorescence was determined with an irrelevant isotype-specific mouse mAb or secondary antibody only.

### ACKNOWLEDGMENTS

The authors wish to thank to Dr. C. Dijkstra and Dr. T. Hunig for providing us with ED7 and R73 mAbs, respectively, and D. Kosec for help in flow cytometry.

*(Received June 28, 1995)*

*(Accepted October 4, 1995)*

### REFERENCES

Adkins B., Mueller C., Okada C., Reichert R., Weissmann I., and Spahgrude G. (1986). Early events in T-cell maturation. Annu. Rev. Immunol. **5**: 325–365.

Barclay A.N., and Mayrhofer G. (1981). Bone-marrow origin of Ia positive cells in the medulla of rat thymus. J. Exp. Med. **153**: 1666–1671.

Bernard G., Zoccola D., Ticchioni M., Breitmayer J.-P., Aussel C., and Bernard A. (1994). Engagement of the CD45 molecule induces homotypic adhesion of human thymocytes through LFA-1/ICAM-3 dependent pathway. J. Immunol. **152**: 5161–5170.

Carlow D.A., van Oers N.S., Teh S.J., and Teh H.S. (1992). Deletion of antigen specific thymocytes by dendritic cells requires LFA-1/ICAM-1 interactions. J. Immunol. **148**: 1595–603.

Caux C., Vanbervliet B., Massacrier C., Azuma M., Okumura K., Lanier L.L., and Bancherau J. (1994). B70/B7-2 is identical to CD86 and is the major functional ligand for CD28 expressed on human dendritic cells. J. Exp. Med. **180**: 1841–1847.

Čolić M., Gašić S., Stojanović N., Popović Lj., and Dujić A. (1992). Phenotypic and ultrastructural characterization of an epithelial cell line established from rat thymic cultures. Immunol. **77**: 201–207.

Čolić M., Ilić V., Tamatani T., Miyasaki M., and Pavlović M.D. (1994). Role of β2 integrins in the binding of thymocytes to rat thymic macrophages. Dev. Immunol. **4**: 65–77.

Damoiseaux J.G.M.C., Dopp E.A., Neefjes J.J., Beelen R.H.J., and Dijkstra C.D. (1989). Heterogeneity of macrophages in rat evidenced by variability in determinants. Two new anti-rat macrophage antibodies against a heterodimer of 160 and 95 kd (CD11/CD18). J. Leuko. Biol. **46**: 556–564.

Damoiseaux J.G.M.C. (1991). Macrophage heterogeneity in the rat. Doctoral thesis, Free University, Amsterdam, pp. 107–121.

Duijvestijn A.M., Sminia T., Kohler Y.G., Janse E.M., and Hoefsmit E.C.M. (1984). Ontogeny of the rat thymus microenvironment: Development of the interdigitating cells and macrophage populations. Dev. Comp. Immunol. **8**: 451–460.

Dustin M.L., and Springer T.A. (1989). T cell receptor cross-linking transiently stimulates adhesiveness through LFA-1. Nature **341**: 619–624.

Fine J.S., and Kruisbeek A.M. (1991). The role of LFA-1/ICAM-1 interactions during murine T lymphocyte development. J. Immunol. **147**: 2852–2859.

Fawcett J., Holness C.L., Needham L.A., Turley., Gatter K.C., Mason D.Y., and Simmons D.L. (1992). Molecular cloning of ICAM-3, a third ligand for LFA-1, constitutively expressed on resting lymphocytes. Nature **360**: 481–484.

Hamblin A.S., and Edgeworth J.D. (1988). Does antigen presentation occur in the thymus. *In*: Thymus update 1. The micro-environment of the human thymus, Kendall M.D., and Ritter, M.A., Eds. (London: Harwood Academic Publishers), pp. 135–153.

Hunig T., Wallny H.-J., Hartley J.K., Lawetzky A., and Tiefenthaler A. (1989). A monoclonal antibody to a constant determinant of the rat T cell antigen receptor that induces T cell activation. Differential reactivity with subset of immature and mature T lymphocytes. J. Exp. Med. **169**: 73–86.

Ilić V., Čolić M., and Kosec D. (In press). Isolation, cultivation and phenotypic characterization of rat thymic dendritic cells. Thymus.

Inaba K., Romani N., Steinman R. (1989). An antigen independent contact mechanism as an early step in T cell-proliferative responses to dendritic cells. J. Exp. Med. **170**: 527–542.

Inaba K.,Witmer-Pack M., Inaba M., Hathcock K.S., Sakuta H., Azuma M., Yagita H., Okumura K., Linsley P.S., Ikehara S., Maramatsu S., Hodes R.J., and Steinman R.M. (1994). The tissue distribution of B7-2 costimulator in mice: Abundant Expression on dendritic cells *in situ* and during maturation *in vitro*. J. Exp. Med. **180**: 1849–1860.

Janossy G., Tidman N., and Selby W.S. (1980). Human T lymphocytes of inducer and suppressor type occupy different microenvironments. Nature **288**: 81–84.

Kampinga J., and Aspinall R. (1990). Thymocyte differentiation and thymic micro-environment development in the fetal rat thymus: An immunohistological approach. In: Thymus update 3. The role of the thymus in tolerance induction, Kendall, M.D., and Ritter, M.A. Eds. (London: Harwood Academic Publisher), pp. 149–186.

Kansas G.S., and Tedder T.F. (1991). Transmembrane signals generated through MHC class II, CD19, CD20, CD39, and CD40 antigens induce LFA-1-dependent and independent adhesion in human B cells through a tyrosine kinase-dependent pathway. J. Immunol. **147**: 4094–4102.

Killen N., Moessner R., Arvieux J., Willis A., and Williams A.F. (1988). The MRC OX-45 antigen of rat leukocytes and endothelium is in a subset of the immunoglobulin superfamily with CD2, LFA-3 and carcinoembrionic antigens. EMBO J. **10**: 3087–3091.

King P.D., and Katz D.R. (1989). Human tonsillar dendritic cell-induced T cell responses: analysis of molecular mechanisms using monoclonal antibodies. Eur. J. Immunol. **19**: 581–587.

King P.D., Batchelor A.H., Lawlor P., and Katz D.R. (1990). The role of CD44, CD45, CD45RO, CD46 and CD55 as potential anti-adhesion molecules involved in the binding of human tonsillar T cells to phorbol 12-myristate 13-acetate-differentiated U-937 cells. Eur. J. Immunol. **20**: 363–368.

Kyewski B.A., Rouse R.V., and Kaplan H.S. (1982). Thymocytes rosettes: Multicellular complex of lymphocytes and bone marrow-derived stromal cells in the murine thymus. Proc. Natl. Acad. Sci. USA **79**: 5646–5650.

Kyewski B.A., Fathman C.G., and Rouse R.V. (1986). Intrathymic presentation of circulating non-MHC antigens by medullary dendritic cells. J. Exp. Med. **163**: 231–246.

Kyewski B. (1988). Unraveling the complexity of intrathymic cell-cell interaction. APMIS **96**: 1049–1060.

Landry D., Doyon L., Poudrier J., Lanfontaine M., Pelletier M., and Montplaisir S. (1990). Accessory function of human thymic dendritic cells in Con A-induced proliferation of autologous thymocyte subset. J. Immunol. **144**: 836–843.

Lepesant H., Reggio H., Pierres M., and Naquet P. (1990). Mouse thymic epithelial cell lines interact with and select CD3$^{low}$ CD4$^+$ CD8$^+$ thymocyte subset trough an LFA-1 dependent adhesion-deadhesion mechanism. Int. Immunol. **2**: 1021–1032.

Lorenz H.M., Harrer T., Lagoo A.S., Baur A., Eger G., and Kalden J.R. (1993). CD45 mAb induces cell adhesion in peripheral blood mononuclear cells via lymphocyte function-associated antigen-1 (LFA-1) and intercellular cell adhesion molecule 1 (ICAM-1). Cell Immunol. **147**: 110–128.

Patarroyo M., Prieto J., Rincon J., Timonen T., Lundberg C., Lindbom L., Asjo B., and Gahmberg C. (1990). Leukcocyte-cell adhesion: A molecular process fundamental in leukocyte physiology. Immunol. Rev. **114**: 67–108.

Pavlović M., Čolić M., Pejnović N., Tamatani T., Miyasaka M., and Dujić A. (1994). A novel anti-rat CD18 mAb triggers lymphocyte homotypic aggregation and granulocyte adhesion to plastic: Different intracellular signaling pathways in resting versus activated thymocytes. Eur. J. Immunol. **24**: 1640–1648.

Pircher H., Brduscha K., Steinhoff U., Kasai M., Zinkernagel R.M., Hengartner H., Kyewski B., and Muller K.-P. (1993). Tolerance induction by clonal deletion of CD4$^+$CD8$^+$ thymocytes *in vitro* does not require dedicated antigen-presenting cells. Eur. J. Immunol. **23**: 669–674.

Prickett T.C., and Hart D.N. (1990). Anti-leukocyte common

(CD45) antibodies inhibit dendritic cell stimulation of CD4 and CD8 T-lymphocyte proliferation. Immunology **69**: 250–256.
Ramsdell F., and Fowlkes B.J. (1990). Clonal deletion verus clonal anergy: The role of the thymus in inducing self tolerance. Science **248**: 1342–1348.
Shortman K., and Vremec D. (1991). Different subpopulations of developing thymocytes are associated with adherent (macrophage) and nonadherent (dendritic) thymic rosettes. Dev. Immunol. **1**: 225–235.
Scheeren R.A., Koopman G., Van der Baan S., Meijer C.J.L.M. and Pals S.T. (1991). Adhesion receptors involved in clustering of blood dendritic cells and T lymphocytes. Eur. J. Immunol. **21**: 1101–1105.
Springer T.A. (1990). Adhesion receptor of the immune system. Nature **346**: 425–434.
Staunton D.E., Dustin M.L., and Springer T.A. (1989). Functional cloning of ICAM-2, cell adhesion ligand for LFA-1 homologous to ICAM-1. Nature **339**: 61–64.
Tamatani T., and Miyasaka M. (1990). Identification of monoclonal antibodies reactive with the rat homologue of ICAM-1, and evidence for a differential involvement of ICAM-1 in the adherence of resting versus activated lymphocytes to high endothelial cells. Int. Immunol. **2**: 165–171.
Tamatani T., Kotani M., and Miyasaka M. (1991a). Characterization of rat leukocyte intergrin, CD11/CD18, by the use of LFA-1 subunit-specific monoclonal antibodies. Eur. J. Immunol. **21**: 627–633.
Tamatani T., Kotani M., Tanaka T., and Miyasaka M. (1991b). Molecular mechanisms underlying lymphocyte recirculation. II. Differential regulation in the interaction between lymphocytes and high endothelial cells. Eur. J. Immunol. **21**: 855–858.
Van Kooyk Y., Van de Wiel-van Kemende P., Weder P., Kuijpers T.W., and Figdor C.D. (1989). Enhancement of LFA-1 mediated cell adhesion by triggering through CD2 and CD3 on T lymphocytes. Nature **324**: 811–813.
Xu H., Friedrichs U., Gieseler R.K., Ruppert J., Ocklind G., and Prters J.H. (1992). Human blood dendritic cells exhibit a distinct T-cell-stimulating mechanism and differentiation pattern. Scand. J. Immunol. **36**: 689–696.

